# *IL-12 RB1* Genetic Variants Contribute to Human Susceptibility to Severe Acute Respiratory Syndrome Infection among Chinese

**DOI:** 10.1371/journal.pone.0002183

**Published:** 2008-05-14

**Authors:** Fang Tang, Wei Liu, Fang Zhang, Zhong-Tao Xin, Mao-Ti Wei, Pan-He Zhang, Hong Yang, Hinh Ly, Wu-Chun Cao

**Affiliations:** 1 Beijing Institute of Microbiology and Epidemiology, Beijing, People's Republic of China; 2 State Key Laboratory of Pathogen and Biosecurity, Beijing, People's Republic of China; 3 Pathology and Laboratory Medicine Department, Emory University School of Medicine, Atlanta, Georgia, United States of America; 4 Affiliated Hospital of APFCP Medical College, Tianjin, People's Republic of China; University of California San Francisco, United States of America

## Abstract

**Background:**

Cytokines play important roles in antiviral action. We examined whether polymorphisms of interleukin (IL)-12 receptor *B*1 (*IL-12RB1)* affect the susceptibility to and outcome of severe acute respiratory syndrome (SARS).

**Methods:**

A case-control study was carried out in Chinese SARS patients and healthy controls. The genotypes of 4SNPs on *IL-12 RB1* gene, +705A/G,+1158T/C, +1196G/C and +1664 C/T, were determined by PCR-RFLP. Haplotypes were estimated from the genotype data using the expectation-maximisation algorithm.

**Results:**

Comparison between patients and close contacts showed that individuals with the +1664 C/T (CT and TT) genotype had a 2.09-fold (95% confidence interval [CI], 1.90–7.16) and 2.34-fold (95% CI, 1.79–13.37) increased risk of developing SARS, respectively. For any of the other three polymorphisms, however, no significant difference can be detected in allele or genotype frequencies between patients and controls. Additionally, estimation of the frequencies of multiple-locus haplotypes revealed potential risk haplotypes (GCCT) for SARS infection.

**Conclusions:**

Our data indicate that genetic variants of *IL12RB1*confer genetic susceptibility to SARS infection, but not necessary associated with the progression of the disease in Chinese population.

## Introduction

Severe acute respiratory syndrome (SARS) is an infectious disease caused by the SARS coronavirus with >8000 cases and 774 deaths reported in 2003 [1]. Much progress has been made in understanding the SARS coronavirus, but its pathogenesis in infected individuals remains unclear [2]. The early reports showed that old age, diabetes mellitus and heart disease were risk factors for adverse prognosis of SARS [3], [4]. Genetic host factors are as well supposed to play an important role, and polymorphisms in several genes have been tested for their association with the infection [5]–[13]. Three studies have investigated role of angiotensin converting enzyme gene in disease pathogenesis or disease outcome with conflicting results [5]–[7]. Two independent studies displayed the significant role of the mannose-binding lectin (*MBL)* genetic polymorphisms in SARS infection [8], [9]. Other candidate genes, such as OAS1 and MxA gene [10], interferon gamma gene [11] and RANTES gene [12], ICAM3 gene [13] were also identified in recent studies. A better understanding of these genes will provide new insights into the disease pathogenesis. This knowledge will also aid in identifying the biomarkers for evaluating the efficacy of vaccination and other interventions.

Interleukin-12 (IL-12) is a cytokine secreted by activated phagocytes and dendritic cells [14]. It plays a pivotal role in promoting Th1-type immune responses and cell-mediated immunity. IL-12 triggers many biological functions: it stimulates the proliferation of activated T- and NK-cells, enhances T- and NK-cell-mediated cytolytic activity, and induces the production of IFN-γ by both T-and NK-cells (Trinchieri 1998). The interferon-γ production induced by IL-12 forms a major link between innate and adaptive immunity [15]. A recent study revealed that interferon-mediated immunopathological events are associated with atypical innate and adaptive immune responses in SARS patients [16]. We thus reasoned that IL-12 might also play a role in resistance to SARS, which is signaled through the direct binding of the IL-12 molecule to its receptor (IL- 12R). IL-12R has been shown to consist of at least two distinct subunits, B1 and B2, that associate to form a high-affinity IL-12R complex expressed on activated T- and NK-cells (17,18). In the current study, we hypothesized that polymorphisms of the *IL-12R* B1 gene might be associated with SARS susceptibility. To test this hypothesis, we conducted a case control study to investigate the association between *IL-12R* B1 genetic polymorphisms and SARS in Chinese individuals.

## Materials and Methods

### Cases and controls

The case and controls were selected from two designated hospitals for SARS in Beijing and Tianjin. During the 2003 SARS epidemic, a total of 132 confirmed SARS patients were reported from these two hospitals, which derived from two completely unrelated super spread events (SSE). All of the patients had been hospitalized for treatment, from which we selected 115 confirmed SARS cases for the study. Three of the remaining patients died and the other 14 patients were unavailable for study, because of the inconvenience of revisiting. The diagnosis was made according to the criteria published by the Chinese Ministry of Public Health, and subsequently confirmed by antibody seroconversion and/or detectable SARS-CoV RNA in respiratory secretions. Two control groups were used, individuals with close contacts with SARS patients (control A) and healthy controls (control B). Control A group consists of 141 subjects who had close contacts with confirmed SARS patients but were not infected, according to criteria that was defined by the World Health Organization [19]. Among these individuals were 110 high-risk health care workers who had been treating SARS patients for a prolonged duration, and 31 individuals who had shared a meal, utensil, residence, ward, vehicle, etc., with SARS patients. Control B group included 155 healthy, unrelated Chinese blood donors, who had blood drawn during July 2001–October 2002. All controls were tested negative for IgG antibody against SARS-CoV prior to being included into the study. All subjects contributed 3ml anti-coagulated blood after written informed consent was obtained. We also used a standard questionnaire to collect demographic information (including age, ethnicity, region of origin, marital status, living environment, education level et al). All subjects were confirmed as genetically unrelated through interviewing. The comorbidity conditions that might have effect on disease susceptibility, such as diabetes mellitus, heart disease, hypertension, asthma, tuberculosis, and malignancy, were traced by reviewing medical records and self reporting. The study protocol was approved by the ethical committee at the National Center for AIDS Prevention and Control.

### IL-12R B1 Genotyping Using PCR-RFLP

Leucocytes were isolated within 12 h of blood collection using Percoll reagent. Genomic DNA was extracted using cell DNA extraction kit (BioDEV Inc. Beijing, China) following the manufacturer's instructions.

We typed the samples for 4 previously reported SNPs that cause missense mutations in the coding sequence of the *IL-12RB1* gene [20] , namely +705A/G (Q214R, NCBI SNP ID:rs 11575934), +1158T/C(M365T, NCBI SNP ID:mrs 375947), +1196G/C (G378R, NCBI SNP ID: rs 401502), and +1664 C/T (P534S, HGBASE database ID: SNP001745641). For +705A/G genotyping, genomic DNA was amplified using the primer set 5′-ggttaagtgactggtgccaag-3′ and 5′- ctcaaaccactggcctcaag-3′. The PCR fragment obtained was restricted with *Bbv* I (New England BioLabs, Beverly, Mass., US) at 37°C. For +1158T/C typing (M365T), 5′-aacaaacgccatctgctacc-3′ and 5′-caacacctctctgggcctta-3 ′, and *Hsp* 92II (Promega, Madison, Wisc., USA) were used at 37°C. For +1196G/C, 5′ -aacaaacgccatctgctacc-3 ′ and 5′-agagtgagaggccacctgag-3 ′ , and *Msp* I (Promega, Madison, Wisc., USA) were used at 37°C. For +1664 C/T, 5′-ggctgtggtagcccagcct-3′ and 5′-ggaagcgcagtgcagtgcatg-3′ were used and restricted with *Bsr* I (New England BioLabs) at 65°C. PCR was carried out in PCR buffer (1.5 m *M* MgCl 2 , 10 m *M* Tris-HCl, pH 9.0, 50 m *M* KCl, 0.1% Triton ® X-100), 100 µ*M* of each dNTP, 25 µ*M* primers and 1 unit of *Taq* polymerase in a final volume of 30 µl. Following an initial denaturation at 95°C for 5 min, samples were subjected to 30 cycles of PCR amplification with denaturation at 95°C for 30 s, annealing at 57–60°C for 30 s and elongation at 72°C for 30 s, followed by a final elongation step at 72°C for 10 min. The reaction products of the PCRs and enzyme restrictions were analyzed on 3% agarose gels (+705A/G, +1158T/C, +1664 C/T) or 8% polyacrylamide gels (+1196G/C). The genotyping of all samples were performed in a blinded manner so that the case or control status was not known. Ten percent of the samples were chosen randomly from each polymorphism for resequencing in order to validate the genotyping method.

### Statistical analysis

Genotype distributions of each SNPs were tested for the Hardy–Weinberg equilibrium by the Chi square test in three groups. The genotype frequencies of the SARS patients and the control subjects were compared, and odds ratios (OR) were calculated by assigning the reference value (1.0) to the homozygous genotype that was more frequent in the control subjects than in the SARS patients. The OR and *P* values were calculated by multivariate logistic regression analysis with age, sex , comorbidity presence and infection source entered as covariates in the model, given the relevance of these potential confounding variables in interpreting the study results. Data were analyzed using the SPSS software (version 10.0, SPSS Inc, Chicago, IL, USA). To correct for multiple testing, we used spectral decomposition (SpD) of matrices of pairwise linkage disequilibrium (LD) between SNPs, which adjusts for multiple testing while taking into account LD among the tested SNPs [21]. This method showed that *P*-values of 0.0227 and below can be considered as statistically significant after correction for multiple testing. The software HAPLOVIEW (version 2.05) was used to construct haplotypes from the genotype data. This program calculates association statistics for multilocus haplotypes in case-control data using the expectation- maximisation algorithm to estimate haplotype odds ratios across multiple categories, giving a likelihood ratio test of homogeneity.

## Results

### Baseline Clinical Characteristics of cases and controls

Table 1 shows the baseline characteristics of SARS patients and two control groups. The mean age was 33.1±11.7 years for SARS cases, 31.6±7.0 years for control A group and 28.4±11.8 years for control B group. 46.7% of SARS patients, 32.5% of control A group and 71% of control B group were male. Sixty-two SARS patients were health care workers. Thirty-nine (33.9%) patients were classified as a severe group, based on their admissions to intensive care units or deaths due to the disease (mean±SD age = 39.45±12.8, 20 male and 19 female). The remaining 76 patients were classified as a mild group (mean±SD age = 29.61±13.5, 46 male and 56 female).

**Table 1:** The demographic characteristics of SARS patients and controls

	SARS patients (n = 115)	Control A (n = 141)	Control B (n = 155)
Ethnic (Han %)	100	100	100
Sex (male %)	32.5	46.7	71.0
Age (mean±SD)	33.1±11.7	31.6±7.0	28.4±11.8
Health care workers (%)	83 (72%)	110 (78%)	0 (0%)
Comorbidity (%)	10 (8.7%)	13 (9.2%)	10 (6.5%)

### Genotype Frequencies of the 4 SNPs of the IL12RB1 gene in SARS Patients and Controls

The Hardy-Weinberg equilibrium test based on the genotype frequency was carried out separately for the three groups of individuals. For control B group, the genotype distributions of the 4 SNPs (+705A/G, +1158T/C, +1196G/C and +1664 C/T) were in Hardy-Weinberg equilibrium, while for patients group, the genotype distribution of +1664 C/T polymorphisms deviate slightly from Hardy-Weinberg equilibrium (data not shown).

The genotyping results were missing from some of the patients, because the DNA samples were not enough for all of the loci detection, or the poor RFLP results, which led to their exclusion from the analysis. Allele frequencies at the four loci were compared (Table 2). +1664 C/T (T) allele is shown to be overrepresented in the group of patients, when compared with those in the control A group (*P*-value 0.044) and control B group (*P*-value 0.056). However, when the Nyholt correction was applied in the screening step, the *P* values turned out to be not significant. The allele frequencies of the other three SNPs (+705A/G, +1158T/C, +1196 G/C) were not significantly different between SARS patients and any of the two control groups, with all the P value to be great than 0.05.

**Table 2:** Allele frequencies of the *IL-12 RB1* 705A/G, 1158T/C, 1196G/C and 1664 C/T SNPs in SARS patients and controls

SNPs	SARS (n = 115)	Control A (n = 141)	Control B (n = 155)	OR^a^ (95% CI)	*P* value^a^	OR^b^ (95% CI)	*P* value^b^
+705A/G							
A	131 (64.2)	137 (60.6)	186 (61.6)	1		1	
G	73 (35.8)	89 (39.3)	116 (38.4)	0.87 (0.89–1.09)	0.224	0.91 (0.62–1.46)	0.171
+1158T/C							
T	124 (64.6)	164 (65.6)	189 (63.0)	1		1	
C	68 (35.4)	86 (34.4)	111 (37.0)	1.10 (0.39–1.70)	0.443	0.81 (0.56–2.41)	0.525
+1196G/C							
G	139(62.6)	165(62.5)	190(63.3)	1		1	
C	83(37.4)	99(37.5)	110 (36.7)	1.01 (0.79–1.71)	0.829	1.06 (0.51–1.31)	0.744
+1664C/T							
C	133 (70.7)	213(80.7)	229(76.3)	1		1	
T	55(29.3)	51(19.3)	71(23.7)	1.92 (1.11–6.06)	0.044	1.35 (1.06–1.70)	0.056

*P*-value and OR (95% CI) were calculated with the use of logistic regression models, adjusted with sex, age, comorbidity presence and infection source.

OR = Odds ratio; CI = confidence interval;

OR ^a^ Patients VS Control A ; OR^b^ Patients VS Control B.

*P* value^a^ Patients VS Control A ; *P* value^b^ Patients VS Control B.

Genotype frequencies at four loci are shown in Table 3. A significant difference in the genotype frequencies between SARS patients and controls was found for the +1664 C/T SNPs (*P*<0.001). The +1664 C/T (CT) and (TT) genotypes were significantly over represented in patients (41.5% and 8.5%, respectively) than in control A group (29.5% and 4.5%, respectively). Compared to the CC genotype, CT and TT genotypes were found to be associated with increased susceptibility to SARS infection with ORs (95% *CI*) of 2.09 (1.90–7.16) and 2.34 (1.79–13.37), respectively. The genotype analysis of the +1664 C/T between SARS patients and control A group yielded different result with those between patients and control B group, when the control B group were compared, no difference of the genotype distribution can be detected. Genotype frequencies of +705 A/G, +1158T/C or +1196 G/C SNPs were not significantly different between SARS patients and each of the two control groups.

**Table 3:** Genotype frequencies of *IL-12 RB1* 705A/G, 1158T/C, 1196G/C and 1664 C/T SNPs in SARS patients and controls groups

SNP	SARS (n = 115)	Control A (n = 141 )	Control B (n = 155 )	OR^a^(95% CI)	*P* value^a^	OR^b^ (95% CI)	*P* value^b^
+A705G							
AA	41 (40.2)	43(38.1)	59(39.1)	1		1	
G A	49(48.0)	51(45.1)	68(45.0)	1.26 (0.20–1.05)	0.171	1.17 (0.95–4.91)	0.223
GG	12 (11.8)	19(16.8)	24(15.9)	0.76 (0.32–2.90)	0.082	0.64 (0.35–3.14)	0.194
+T1158C							
TT	40 (41.7)	56(44.8)	59(39.3)	1		1	
TC	44 (45.8)	52(41.6)	71(47.3)	1.29 (0.94–3.44)	0.105	0.90 (0.53–2.68)	0.137
CC	12 (12.5)	17 (13.6)	20(13.3)	0.71 (0.39–3.97)	0.219	0.84 (0.44–5.01)	0.166
+G1196C							
GG	44 (39.6)	53(40.2)	59 (39.3)	1		1	
GC	52 (46.8)	59(44.7)	72(48.0)	1.19 (0.71–3.07)	0.712	0.85 (0.25–6.10)	0.221
CC	15(13.5)	20(15.2)	19(12.7)	0.86 (0.74–3.83)	0.328	1.13 (0.39–6.95)	0.417
+C1664T							
CC	47(50.0)	87(65.9)	88(58.7)	1		1	
CT	39(41.5)	39(29.5)	52(34.7)	2.09 (1.90–7.16) *	0.020*	1.45 (1.16–2.78)	0.047
TT	8(8.5)	6(4.5)	10 (6.7)	2.34 (1.79–13.37) *	0.022*	1.47 (0.26–2.26)	0.060

*P*-value and OR (95% CI) were calculated with the use of logistic regression models, adjusted with sex, age, comorbidity presence and infection source.

OR = Odds ratio; CI = confidence interval;

OR ^a^ Patients VS Control A ; OR^b^ Patients VS Control B.

*P* value^a^ Patients VS Control A ; *P* value^b^ Patients VS Control B.

* Significant *P* value after correction by Nyholt method

### Association between genotype frequencies of the 4 SNPs and the severity of SARS

SARS patients were then divided into two subgroups according to the severity of the disease: advanced subgroup (i.e., patients with moderate or advanced lung disease) and mild subgroup. Multiple regression models were re-established for each SNP, with age, sex and the source of infection hospital as covariates. Association with the severity of SARS infection was then estimated by comparing the genotype frequencies and allele frequencies of all the SNPs between the two groups of SARS patients. The final model building disclosed prominent associations between advanced disease with carrying +1664 C/T (CT) and (TT) genotype (*P* = 0.037). However, this was found not to be significant after Nyholt correction was applied to the analysis. Again, there were no significant differences in the genotype and allele distributions of the +705 A/G , +1158T/C or +1196 G/C SNPs between those with mild disease and those with advanced disease (data not shown).

### Haplotype analysis

Pairwise linkage disequilibrium (LD) analysis of the four SNPs showed strong LD among +705 A/G, +1158T/C and +1196G/C SNPs (D′ = 0.90–0.98) and modest LD value between +1664 C/T and one of the other three SNPs (D′ = 0.64–0.81), thus revealing two common haplotypes (A-T-G-C (705-1158-1196-1664) and G-C-C-T). To investigate if a particular haplotype constituted by these SNPs was associated with the disease, haplotype frequencies were estimated and association analysis was performed using multiple logistic regression with age, sex and infection source being the covariants. As shown in Table 4, the frequency of GCCT haplotype in SARS patients (26.6%) was significantly increased when compared to the control A group (11.1%), but when compared to the control B group, the proportions of this haplotype in the two groups were not different (26.6% in SARS patients vs. 22.3% in controls).

**Table 4:** Estimated frequencies of haplotypes constituted by 705A/G, 1158T/C, 1196G/C and 1664 C/T SNPs of *IL-12 RB1* in SARS patients and controls

Haplotype	SARS patients N(%)	Control A N (%)	Control B N (%)	OR^a^ (95% CI)	*P* value^a^	OR^b^ (95% CI)	*P* value^b^
ATGC	111 (59.0)	131(58.0)	182(60.7)	1		1	
GCCT	50 (26.6 )	25(11.1)	67(22.3)	2.31 (1.72–8.47) *	0.011*	1.14 (0.81–3.10)	0.443
GCCC	15(7.9)	52(23.0)	28(9.3)	0.29 (0.07–0.63) *	0.017*	0.90 (0.13–1.97)	0.602
Others^c^	12 (6.4)	18 (8.0)	23(7.7)	0.76 (0.39–2.01)	0.314	0.92 (0.44–4.19)	0.580

*P*-value and OR (95% CI) were calculated with the use of logistic regression models, adjusted with sex, age, comorbidity presence and infection source.

OR = Odds ratio; CI = confidence interval;

a OR of Patients VS ControlA ;

b OR of Patients VS Control B.

c Haplotypes with frequencies <5%.

* Significant *P* value after correction by Nyholt method

## Discussion

*IL-12R* has previously been reported to be associated with infectious diseases such as tuberculosis, hepatitis B virus infection, and parvovirus infection [22]–[24], revealing its potential role of function in host defense against microbial infections. We genotyped 4 SNPs (+705A/G, +1158T/C , +1196G/C and +1664 C/T) that have been alleged to cause missense mutations of the *IL-12 RB1* gene in SARS patients , close contacts and healthy controls. These four SNPs (one novel) have been confirmed to cause missense variants in the extracellular coding sequence of the *IL12R*Β*1*gene. These four SNPs lie within 6.2-kb span of genomic DNA on chromosome 19p13.1. It is suggested that one or more of the four missense variants could affect the quality (or quantity) of the gene products and cause mild functional impairment in the receptor's responsiveness to IL-12 [25]. The results show that individuals with 1664 C/T (CT) and (TT) genotype were at increased risk of susceptibility to SARS infection. When only severe patients were considered, the significance disappeared, in comparison with the mild patients. We also estimated the frequencies of *IL-12 RB1* haplotypes, which composed of 4 polymorphic alleles, and identified risk haplotype (GCCT) that might confer genetic susceptibility to SARS infection. In agreement with this finding, the +1664 C/T (T) allele was among the risk haplotypes GCCT, whereas the alternative C allele was among the protective haplotype (GCCC). These findings support the association between *IL12B1* polymorphism and SARS infection, but not necessary the disease outcome in Chinese population.

It is interesting to note that these associations only exist when close contacts were used as a control group. When healthy blood donors were used as controls, no association could be detected. We reasoned that genetic difference between the two control groups might partly account for the distinct observations with regard to SARS susceptibility, i.e. close contacts might be more immunogenetically resistant to SARS infection than healthy controls. In this case, close contacts might serve as more suitable controls healthy individuals, who had never been exposed to SARS-CoV. It's as well intriguing that the pattern of polymorphism in +1664 C/T in SARS patients deviated slightly from Hardy-Weinberg equilibrium. As noted in the previous study[26], detection of HW disequilibrium at SNP loci in the case sample could be an indicator that a disease/susceptibility gene is within the region. There are also other possibilities indicated, like that a pseudo-SNP is present, DNA contamination, or a genotyping error, however, these latter possibilities are unlikely since we have duplicated the genotyping assay for all the samples with ambiguous results through sequencing. This also can be argued from the observed perfect adherence to Hardy-Weinberg disequilibrium from control group. The chance of subgroup is minor, since all the subjects were Chinese Han population without consanguineous relationship. Taken together, we suggested this deviation was from the correlation between genetic variant and disease, instead of false association.

SARS pathogenesis is less well understood, yet it is a vital issue in the disease management. It has been suggested that genetic variations of the host and/or the virus may account for the individual difference in the severity of the disease. Studies have addressed some of the host genes that might play a role in disease development [5], [7]. However, all of the available studies have failed to take viral factor(s) into consideration. These studies thus cannot exclude the possible false association caused by the differentiated viral adaption in humans. The SARS patients in the current study were mostly health care workers in two SARS-designated hospitals. Epidemiological investigations have clearly defined detailed transmission among those patients, showing each of the group came from one super spread chain arising from one infection source. In the multiple logistic analysis, we used the infection source as one of the covariants, and by doing so, we could technically exclude the potential confounding effect that might be caused by viral factor(s). Advanced age and presence of comorbidity are other two factors that might influence the disease susceptibility[3], [4], which were excluded from potential confounding by multiple variants analysis.

It should be mentioned not all of the subjects were genotyped due to the inadequacy of DNA samples. Especially for the +C1664T SNP, different percentages of genotypes were missing from SARS, Control A, and Control B groups (18.2%, 6.4% and 3.2% respectively). One can reasonably argue there was another SNP that accounted for the increased fraction of subjects without the defined genotypes in the SARS patient group. It can also be argued that the significant SNPs actually are not functional variants of the IL-12RB1 protein, but instead it is presumably in linkage disequilibrium with a potential functional variant(s) in other loci of the gene. Presently 15 coding, nonsynonymous SNPS are listed in the Entrez database dbSNP, out of a total of 274 known human SNPs for IL12RB1, however, the genotyping for other SNPs in small group of the current population showed MAF (minor allele frequency) of less than 5% (unpublished data). We thus propose the lack of a complete analysis of the SNPS in this gene can pose no severe damage to the study. Still we could not formally exclude possible additional causal variation(s) elsewhere in the gene. The sequencing of the exon and the promoter regions of the IL12RB1 gene from the SARS patient cohort could be helpful in identifying other SNPs which could alter the gene expression level. It would also be warranted to examine whether the same association is observed in other ethnic groups. On the other hand, a relatively small sample size inevitably resulted in limitations of the power to detect significant association in disease outcome. As such, the negative results on SNPs for disease outcome revealed in this study do not necessarily exclude their association with SARS.

In one previous study, marked elevation of IL-12 in plasma of SARS patients had been observed for at least 2 weeks after disease onset[27], Since IL-12 can induce the production of IFN-γ and other Th1 cytokines with suppression of the Th2 pathway [28], [29].the observed early elevation of IL-12 could be causative of the SARS-CoV-induced activation of Th1 cells and NK cells, release of chemokines such as IL-8, and results in pulmonary inflammation [30]. One could then hypothesize that decreased IL-12R activity might predispose to infection but protect from severe disease manifestations. However, the data presented here appear to suggest that IL-12R activity is required for protection from severe disease, although the association between genetic polymorphisms and severe disease was no longer significant after multiple test correction was applied to the analysis. Therefore the actual role of IL12 in the protection from or susceptibility to severe disease still need more evidence from animal experiments.

In summary, our data support the association between *IL-12 RB1* polymorphism and SARS. These genetic variations might predispose individuals to SARS infection by diminishing receptor responsiveness to IL-12, leading to partial dysfunction of interferon-γ-mediated immunity. Further genetic and biologic studies of these genetic variants could also render valuable insights into SARS susceptibility and pathogenesis.
